# Palliative chemotherapy outcomes in patients with ECOG-PS higher than 1

**DOI:** 10.3332/ecancer.2018.831

**Published:** 2018-04-30

**Authors:** Rafael Caires-Lima, Karolina Cayres, Bruno Protásio, Inacelli Caires, Júlia Andrade, Lucila Rocha, Tiago Kenji Takahashi, Paulo M Hoff, Gilberto de Castro, Milena Perez Mak

**Affiliations:** Medical Oncology, Instituto do Cancer do Estado de Sao Paulo, Sao Paulo 01246-000, Brazil

**Keywords:** palliative chemotherapy, supportive care, prognosis, poor performance status, end-of-life care

## Abstract

**Purpose:**

Although patients with incurable disease and Eastern Cooperative Oncology Group performance status (ECOG-PS ≥ 2) are underrepresented in clinical trials, they are frequently offered palliative chemotherapy (pCT) in daily clinical practice in order to improve symptoms and quality of life. In this case-control retrospective analysis, our goal was to identify factors associated with poorer survival and lack of benefit of pCT in this population.

**Patients and methods:**

We evaluated 2,514 patients who died between August 2011 and July 2012 in an academic cancer care institution and its hospice. A total of 301 patients with solid tumours and ECOG-PS ≥ 2 at prescription of pCT were selected for this case-control retrospective analysis. Cases were defined as patients who survived less than 90 days after the first cycle of first line pCT, and controls were those who had a longer survival.

**Results:**

142 cases and 159 controls were included. Cases were more likely to experience grade ≥ 3 toxicity (43% versus 28%; *p* = 0.005), die of toxicity (16% versus 6%; *p* < 0.001) and not be offered best supportive care (BSC) only (47% versus 71%; *p* < 0.001). Median overall survival was 204 among controls and 34 days in cases (hazard ratio = 0.177; 95%, confidence interval = 0.015–0.033, *p* < 0.001). Logistic regression analysis identified ECOG-PS > 2 (odds ratio (OR) = 2.3, *p* = 0.044) and serum creatinine (sCr) > 1 mg/dL (OR = 11.2, *p* < 0.001) as independent predictors of 90-day mortality.

**Conclusions:**

The independent predictors of short survival (less than 3 months) after initiation of pCT in this population were ECOG-PS > 2 and elevated sCr. Therefore, patient selection is crucial, as pCT may be deleterious in ECOG-PS ≥ 2 pts.

## Background

Throughout the years, palliative chemotherapy (pCT) has evolved and provided patients with metastatic cancer higher survival rates [2, 8]. In addition, improvement in supportive care and drug development have facilitated adverse events management and yielded better tolerance to chemotherapy. Hence, pCT has become the standard treatment for cancer patients with unresectable or metastatic disease. In this scenario, the main goals of treatment are to provide symptomatic relief as well as prolong survival with manageable toxicity in a number of diseases.

Previous studies have shown that the Eastern Cooperative Oncology Group performance status (ECOG-PS) is a powerful prognostic factor in patients submitted to pCT [10–12, 16, 19]. Poor performance status patients (ECOG-PS ≥ 2) usually tolerate pCT poorly, with increased toxicity compared to patients with ECOG-PS 0–1 [18, 20]. With few exceptions, this patient population is underrepresented in randomised clinical trials and the actual benefit of pCT in this setting in unknown. In this scenario, the choice of BSC can be an acceptable approach [13, 25]. Besides, it should be emphasised that some reports have shown that early introduction of palliative care integrated to standard oncologic treatment reflected in improvement in quality of life, mood and survival [3, 21, 24].

In daily practice, however, unselected patients with ECOG-PS ≥ 2 are often treated with pCT, as a tool to provide patients some gain in terms of symptom palliation and even a doubtful gain in survival [15]. Clinicians’ estimates of patient’s survival can be flawed and subjected to personal experience [6]. In order to improve such forecasting, numerous scores have been developed. However, most of these scores were validated in patient population under BSC alone [14, 22]. This underscores the need to identify clinical characteristics of poor performance status patients who derive benefit from pCT, avoiding overtreatment and providing patients with the best palliative treatment.

The aim of this study was to identify prognostic factors associated with poorer survival and lack of benefit of pCT in this subset of patients.

## Materials and methods

### Endpoints

A case-control retrospective analysis was conducted. The primary endpoint was to identify prognostic factors associated with short overall survival (less than 3 months) in patients with unresectable or metastatic incurable solid tumours who presented ECOG-PS greater than or equal to two at the time of first line pCT. Secondary endpoints were to assess the percentage of patients with improvement in ECOG-PS after pCT, incidence of higher than grade 2 toxicity, time from the last day of pCT to death, percentage of patients referred for exclusive BSC, percentage of patients who died due to toxicity and to estimate the median overall survival in both case and control populations.

## Patient selection

Three hundred and one consecutive patients with unresectable or metastatic solid tumours, who presented ECOG-PS greater than or equal to two at the beginning of first line chemotherapy, were identified from 2,514 patients who died between August 2011 and July 2012 in a public Brazilian tertiary cancer care institution (Instituto do Cancer do Estado de Sao Paulo) or its hospice.

Cases were defined as patients who survived less than 90 days after the first cycle of first line pCT, and controls were those who had a longer survival.

Exclusion criteria were patients who died during the same period, but received curative, neoadjuvant or adjuvant chemotherapy, tyrosine kinase inhibitor in first-line treatment, hormonal therapy or perioperative chemotherapy for liver metastasectomy. Patients with germ cell tumours were excluded from enrolment. Patients with missing data in their electronic medical records were also excluded.

## Data extraction and definitions of terms

Data of eligible patients were collected from electronic medical records. All researchers involved were trained in order to provide uniform data extraction. Patients’ characteristics analysed were age, sex, date of diagnosis, ECOG-PS at beginning of treatment, primary tumour site, weight loss more than 10%, oedema, dyspnoea, pain requiring opioid, altered mental status, cardiovascular comorbidities, diabetes mellitus, dementia, use of enteral feeding, serum haemoglobin, creatinine, ionised calcium, albumin and c-reactive protein, previous admission and first cycle of pCT in-hospital. Overall survival was defined as time between the day of the first pCT cycle and death. Toxicities were graded based on National Cancer Institute–common terminology criteria for adverse events (NCI-CTC AE) version 4.03. All patients who underwent pCT also received BSC as part of their treatment.

## Statistical analysis

Frequencies were compared by chi-square test or Fisher exact test. Risks were estimated by odds ratios (ORs) and logistic regression analysis to determine possible prognostic factors. Overall survival was calculated by Kaplan–Meier method and curves were compared using log-rank test. All analyses were performed by MedCalc (MedCalc, Mariakerk, Belgium), version 11.5.1.0 and SPSS version 18 (SPSS, Chicago, IL). This study was submitted and approved by the local Ethics Research Committee.

## Results

In this analysis, 142 cases and 159 controls were included. Patients’ characteristics are summarised in Table 1. Patients included as cases were slightly younger (58 versus 63 years, *p* = 0.09). Gender was equal between both groups, with female patients frequency of 49% and 50% [*p* = not significant (NS)] in each group, respectively. Cases presented more frequently with ECOG-PS 3 or 4 when initiating pCT than controls (*p* = 0.03; chi-square). Gastrointestinal (31%) and lung cancers (17%) were the most frequent primary tumours in both groups (*p* = 0.077; chi-square).

Significant adverse prognostic factors found in the univariate analysis included older age (>60 years old, OR = 1.7; 95% confidence interval (CI) = 1.0–2.6) and higher PS (ECOG > 2, OR = 1.9; 95% CI = 1.2–3.1), as expected. As found in other reports [14,22], weight loss >10% (OR = 1.8; 95% CI = 1.1-2.8), low haemoglobin (OR = 2.6; 95% CI = 1.6-4.2), low albumin (OR = 2.7; 95% CI = 1.5–5.1), high C-reactive protein (OR = 8.6;95% CI = 1.0–72.9), elevated serum creatinine (sCr) (OR = 2.8; 95% CI = 1.6–5.0), altered mental status (OR = 4.2; 95% CI = 1.4–13.2) and in-hospital pCT (OR = 3.2; 95% CI = 1.9–5.2) were also related to poor survival [5]. Curiously, diabetes mellitus (OR = 0.41, 95% CI = 0.19–0.86) and cardiovascular comorbidities (OR = 0.57, CI = 0.35–0.91) were also associated with longer survival. Prognostic factors are given in Table 2.

Logistic regression analysis identified ECOG-PS > 2 (OR = 2.3, *p* = 0.044) and elevated sCr > 1 mg/dL (OR = 11.2, *p* = 0.0002) as independent prognostic factors of survival less than 3 months. Cardiovascular comorbidities also persisted as predictor of longer survival in this analysis (OR = 0.34, *p* = 0.0159) (Table 2).

Analysing outcomes after pCT (Table 3), cases were more likely to experience grade three or more toxicity (43% versus 28%; *p* = 0.005). However, febrile neutropenia was not statistically different between groups (11.3% versus 7.6%; *p* = 0.36). Controls had a more expressive improvement in ECOG-PS (27.2% versus 1.41%; *p* < 0.0001). Cases experienced more toxic deaths (16% versus 6%; *p* = 0.0007) and were less likely to be kept under BSC alone after failure of first line chemotherapy and beyond (47% versus 71%; *p* < 0.0001). More patients in case group also received pCT in their last month of life (71.1% versus 12.6%; *p* < 0.0001), with a median time from last pCT to death of 22.5 days in cases and 76.5 days in the control group (*p* < 0.001).

**Figure 1.**
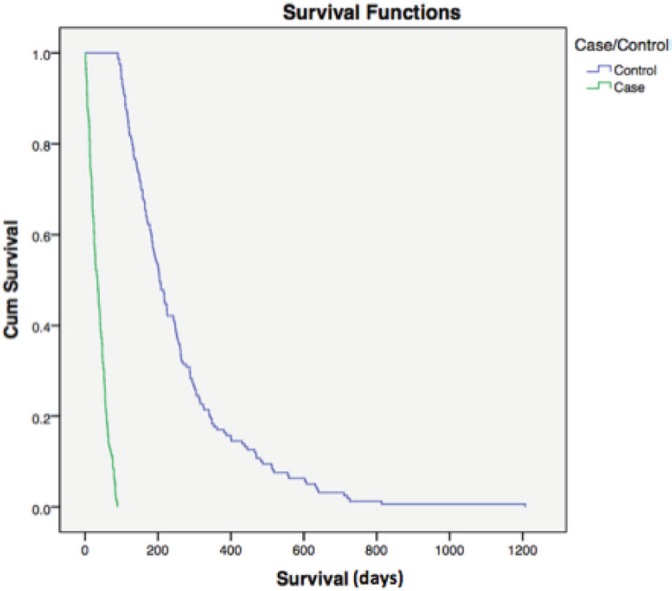
Overall survival case versus controls. Median overall survival was 34 and 204 days among cases and controls, respectively (HR = 0.177; 95% CI = 0.015–0.033, p < 0.0001). Cum = cumulative.

**Figure 2.**
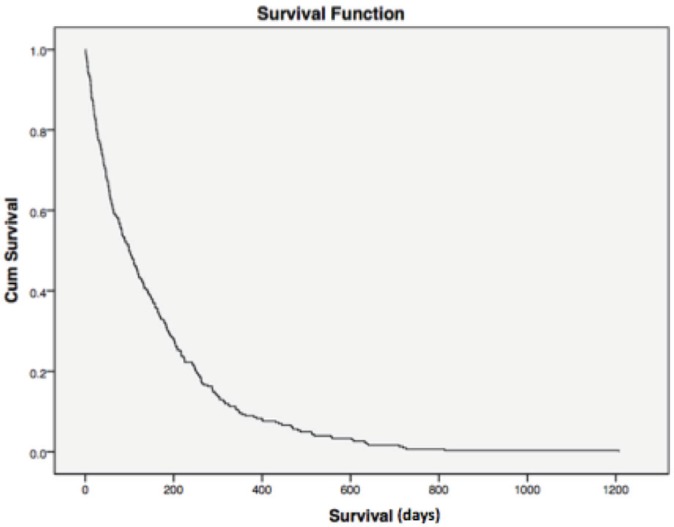
Overall survival. For all patients, median overall survival was 99 days (1–1207), demonstrating the poor prognosis of this population of poor-PS patients overall. Cum = cumulative.

Median overall survival was 34 and 204 days between cases and controls, respectively (hazard ratio (HR) = 0.177; 95% CI = 0.015–0.033, *p* < 0.0001) (Figure 1). For all patients, median overall survival was 99 days (1–1207), demonstrating the poor prognosis of this population of poor-PS patients overall (Figure 2).

## Discussion

In this study, we investigated the prognostic factors associated with poor survival after first line pCT. We hypothesised that a survival shorter than 3 months after initiating first line pCT implies its lack of benefit and determining the factors associated with it could aid physicians to better select patients and avoid unnecessary toxicity.

As we could see, age older than 60 years, ECOG-PS higher than 2, weight loss higher than 10%, serum haemoglobin less than 10 g/dL, serum albumin less than 3 g/dL, sCr higher than 1 mg/dL, serum C-reactive protein higher than 5 mg/L, altered mental status and in-hospital first cycle of pCT were identified as prognostic factors in univariate analysis. However, only ECOG-PS > 2 and sCr higher than 1 mg/dL persisted after logistic regression.

Interestingly, hypercalcaemia, a known poor prognostic factor [17], was not found to be related to short survival in this specific population. A low prevalence was found (7%), reflecting two possible scenarios: either CT-naïve patients who were not amenable for systemic treatment due to hypercalcaemia related symptoms or a late, final disease event.

Another unexpected finding was the association of cardiovascular comorbidity to longer overall survival. This could reflect the benefits of concomitant medications (statins, metformin and aspirin) usually administered to this patient population, which could represent bias of a retrospective study or collinearity effect in logistic regression analysis [1,4].

From all 2,514 patients who died in this period, those with ECOG-PS higher than one at the beginning of pCT who survived less than 3 months (cases) represented only 5.64%. Only 124 patients (4.93% of all cases) received the last dose of chemotherapy in their last month of life. This is within the acceptable threshold (10%) for chemotherapy administration in the end-of-life [9].

Decision making towards continuity of chemotherapy treatment is influenced not only by clinical factors, but also by clinician’s personal experience and patient preference [7]. However, administration of pCT in patients with poor prognosis can lead to delayed hospice referral, increased invasive procedures, such as mechanical ventilation, and lower likelihood of dying in the preferred place of death [23].

## Conclusion

According to our results, pCT needs to be prescribed with caution in patients with ECOG-PS greater than or equal to 2, since it seems to offer no benefit in overall survival, as well as possibly worsen the quality and length of life. Here, we identified ECOG-PS > 2 and elevated sCr as possible adverse prognostic factors, which, if present, should alert clinicians to avoid routine pCT administration.

## Conflicts of interest

The authors have no conflicts of interest to declare.

## Figures and Tables

**Table 1.** Patients’ characteristics.

	Cases	Controls	*p* value
	***N* = 142 (%)**	***N* = 159 (%)**	0.1–0.7 nM
**Age (years)**			0.541
18–40	13 (9.2)	15 (9.4)	
40–60	59 (41.5)	55 (34.6)	
> 60	70 (49.3)	89 (56)	
Median	58 (17–86)	63 (18–87)	
**Sex**			0.850
Male	73 (51.4)	80 (50.3)	
Female	69 (48.6)	79 (49.7)	
**ECOG-PS**			0.006
ECOG-2	67 (47.2)	101 (63.5)	
ECOG-3	65 (45.8)	56 (35.2)	
ECOG-4	10 (7)	2 (1.3)	
**Primary**			0.016
Lung			
NSCLC	17 (12)	25 (15.8)	
SCLC	6 (4.2)	5 (3.1)	
Gastrointestinal			
Oesophageal/stomach	26 (18.3)	17 (10.6)	
Pancreas/biliary/liver	19 (13.4)	13 (8.2)	
Colorectal	17 (12)	24 (15.1)	
Other gastrointestinal	1 (0.7)	10 (6.3)	
Breast	9 (6.3)	4 (2.5)	
Head and neck	4 (2.8)	14 (8.8)	
Urinary	9 (6.3)	15 (9.4)	
Gynaecological	4 (2.8)	20 (12.6)	
Unknown primary	10 (7)	5 (3.1)	
Others	11 (7.7)	7 (4.4)	
**Metastatic at diagnosis**	95 (67)	103 (65)	0.699
**Weight loss > 10%**	80 (56)	68 (43)	0.031
**Anaemia**	116 (82)	108 (68)	0.031
**Hypoalbuminemia**	62 (44)	45 (28)	0.011
**High CRP**	109 (77)	76 (48)	<0.001
**High creatinine**	42 (30)	20 (13)	0.001
**Hypercalcaemia**	10 (7)	8 (5)	0.113
**Oedema**	31 (22)	34 (21)	0.566
**Dyspnoea**	39 (28)	35 (22)	0.273
**Opioid use**	83 (59)	86 (54)	0.446
**Altered mental status**	14 (10)	4 (3)	0.007
**Previous admission**	55 (39)	62 (39)	0.963
**Tube feeding**	21 (15)	19 (12)	0.499
**CVD**	44 (31)	70 (44)	0.066
**DM**	11 (8)	27 (17)	0.055
**Dementia**	-	5 (3)	0.103
**In-hospital CT**	68 (48)	36 (23)	<0.001

NSCLC = non-small cell lung cancer; SCLC = small cell lung cancer; CRP = C-reactive protein; CVD = cardiovascular disease; DM = diabetes mellitus and CT =chemotherapy

**Table 2.** Prognostic factors (primary endpoint).

	Univariate analysis OR (CI 95%)	Logistic regression
Age > 60 years	1.7 (1.00–2.60)	NS
ECOG > 2	1.94 (1.20–3.10)	OR 2.3 (*p* = 0.044)
Weight loss > 10%	1.8 (1.10–2.80)	NS
Haemoglobin < 10 g/dL	2.59 (1.61–4.16)	NS
Albumin < 3 g/dL g/	2.72 (1.45–5.11)	NS
CRP ≥ 5 mg/L	8.60 (1.01–72.93)	NS
Creatinine > ULN	2.86 (1.58–5.17)	OR 11.2 (*p* = 0.0002)
Creatinine clearance < 60 ml/min	1.77 (1.07–2.92)	NS
Altered mental status	4.23 (1.36–13.19)	NS
CVD	0.57 (0.35–0.91)	OR = 0.34, *p* = 0.0159
DM	0.41 (0.19–0.86)	NS
In-hospital CT	3.18 (1.93–5.23)	NS

CRP = C-reactive protein; ULN = upper level of normality; CVD = cardiovascular disease; DM = diabetes mellitus; CT =chemotherapy; NS = not significant

**Table 3.** Secondary endpoints.

	Cases *N* = 142 (%)	Controls *N* = 159 (%)	*p* value
Toxicity > G2*	43%	28%	0.005
Febrile neutropenia	11.3%	7.6%	0.360
Response rate	10.6%	41.3%	<0.001
Improved ECOG	1.4%	27.2%	<0.001
Referred to exclusive BSC	47%	71%	<0.001
Death due toxicity	16%	6%	<0.001
Median time from last CT to death (days)	22.5 (0–77)	76.5 (4–1103)	<0.001
CT in last month of life	71.1 %	12.6%	<0.001
Median overall survival	34 days	204 days	<0.001

* Toxicity graded by (NCI-CTC AE) version 4.0.

BSC = Best supportive care; CT = Chemotherapy
